# Heating Metals in Vacuo by the Electric Current

**Published:** 1880-01

**Authors:** T. A. Edison


### ARTICLE VI.

### *Heating Metals in Vacuo by the Electric Current.*

A very interesting paper, by Mr. T. A. Edison, was read before the American Association at Saratoga the other day.

"In the course of my experiments on electric lightning," says the author, "I have developed some striking phenomena arising from the heating of metals by flames and by the electric current, especially wires of platinum and platinum alloyed with iridium. These experiments are still in progress.

"The first fact observed was that platinum lost weight when heated in a flame of hydrogen, that the metal colored the flame green, and that these two results continued until the whole of the platinum in contact with the flame had disappeared.

"A platinum wire, twenty-thousandths of an inch in diameter, was wound in the form of a spiral one-eighth of an inch in diameter and half an inch in length. The two ends of the spiral were secured to clamping posts, and the whole apparatus was covered with a glass shade. Upon bringing the spiral to incandescence for twenty minutes that part of the globe in line with the sides of the spiral became slightly darkened; in five hours the deposit became so thick that the incandescent spiral could not be seen through the deposit. This film, which was most perfect, consisted of platinum, and I have no doubt but that large plates of glass might be coated economically by placing them on each side of a large sheet of platinum, kept incandescent by the electric current. This loss in weight, together with the deposit upon the glass, presented a very serious obstacle to the use of metallic wires for giving light by incandescence, but this was easily surmounted after the cause was ascertained. I coated the wire forming the spiral with the oxide of magnesium by dusting upon it finely powdered acetate of magnesium. While incandescent the salt was decomposed by the heat, and there remained a strongly adherent coating of the oxide. This spiral so coated was covered with a glass shade and brought to incandescence for several minutes; but instead of a deposit of platinum upon the glass there was a deposit of the oxide of magnesia. From this and other experiments I became convinced that this effect was due to the washing action of the air upon the spiral; that the loss of weight in and the coloration of the hydrogen flame was also due to the wearing away of the surface of the platina, by the attrition produced by the impact of the stream of gases upon the highly incandescent surface, and not to volatilization, as commonly understood.

"I will now describe other and far more important phenomena observed in my experiments.

"If a short length of platinum wire, one-thousandth of an inch in diameter, be held in the flame of a Bunsen burner, at some part it will fuse and a piece of the wire will be bent at an angle by the action of the globule of melted platinum; in some cases there are several globules formed simultaneously, and the wire assumes a zigzag shape.

"With a wire four-thousandths of an inch in diameter this effect does not take place, as the temperature cannot be raised to equal that of the smaller wire owing to the increased radiating surface and mass. After heating, if the wire be examined under a microscope, that part of the surface which has been incandescent will be found covered with innumerable cracks. If the wire be placed between clamping posts, and heated to incandescence for twenty minutes by the passage of an electric current the cracks will be so enlarged as to be seen with the naked eye; the wire under the microscope presents a shrunken appearance, and is full of deep cracks. If the current is continued for several hours these effects will so increase that the wire will fall to pieces.

"This disintegration has been noticed in platina long subjected to the action of a flame, by Prof. John W. Draper. The failure of the process of lighting invented by the French chemist, Tessiè-du-Motay, who raised sheets of platinum to incandescence by introducing them into a hydrogen flame, was due to the rapid disintegration of the metal. I have ascertained the cause of this phenomenon, and have succeeded in eliminating that which produces it, and in doing so have produced a metal in a state hitherto unknown, and which is absolutely stable at a temperature where nearly all substances melt or are consumed, a metal which, although originally soft and pliable, becomes as homogeneous as glass and as rigid as steel. When wound in the form of a spiral it is as springy and elastic when at the most dazzling incandescence as when cold, and cannot be annealed by any process now commonly known.

"For the cause of this shrinking and cracking of the wire is due entirely to the expansion of the air in the mechanical and physical pores of the platinum, and the contraction upon the escape of the air. Platinum as sold in commerce may be compared to sandstone in which the whole is made of a great number of particles with many air spaces. The sandstone upon melting becomes homogeneous and no air spaces exist. With platinum or any metal the air spaces may be eliminated and the metal made homogeneous by a very simple process. This process I will now describe. I had made a large number of platinum spirals, all of the same size and from the same quality of wire; each spiral presented to the air a radiating surface of three and one-sixteenths of an inch; five of these were brought by the electric current up to the melting point, the light was measured by a photometer, and the average light was equal to four standard candles for each spiral just at the melting point. One of the same kind of spirals was placed in the receiver of an air pump and the air exhausted to two millimeters; a weak current was then passed through the wire to warm it slightly for the purpose of assisting the passage of the air from the pores of the metal into the vacuum. The temperature of the wire was gradually augmented at intervals of ten minutes until it became red. The object of slowly increasing the temperature was to allow the air to pass out gradually and not explosively. Afterward the current was increased at intervals of fifteen minutes. Before each increase in the current the wire was allowed to cool, and the contraction and expansion at these high temperatures caused the wire to weld together at the points previously containing air. In one hour and forty minutes this spiral had reached such a temperature without melting that it was giving a light of twenty-five standard candles, whereas it would undoubtedly have melted before it gave a light of five candles had it not been put through the above process. Several more spirals were afterward tried, with the same result. One spiral which had been brought to these high temperatures more slowly gave a light equal to thirty standard candles. In the open air this spiral gave nearly the same light, although it required more current to keep it at the same temperature.

"Upon examination of these spirals, which had passed through the vacuum process, by the aid of a microscope, no cracks were visible; the wire had become as white as silver, and had a polish which could not be given it by any other means. The wire had a smaller diameter than before treatment, and it was exceedingly difficult to melt in the oxyhydrogen flame, as compared with untreated platinum; it was found that it was as hard as the steel wire used in pianos, and that it could not be annealed at any temperature.

"My experiments with many metals treated by this process have proved to my satisfaction, and I have no hesitation in stating, that what is known as annealing of metals to make them soft and pliable is nothing more than the cracking of the metal. In every case where a hard drawn wire had been annealed a powerful microscope revealed myriads of cracks in the metal.

"Since the experiments of which I have just spoken, I have, by the aid of Sprengel mercury pumps, produced higher exhaustions, and have, by consuming five hours in excluding the air from the wire and intermitting the current a great number of times, succeeded in obtaining a light of eight standard candles from a spiral of wire with a total radiating surface of 1–32 of an inch, or a surface about equal to a grain of buckwheat.

"With spirals of this small size which have not passed through the process the average amount of light given out before melting is less than one standard candle. Thus I am enabled by the increased capacity of platinum to withstand high temperatures, to employ small radiating surfaces, and thus reduce the energy required for candle-light. I can now obtain eight separate jets, each giving out an absolutely steady light, and each equal to sixteen standard candles, or a total of one hundred and twenty-eight candles, by the expenditure of thirty thousand foot pounds of energy, or less than one horse power.

"As a matter of curiosity I have made spirals of other metals, and excluded the air from them in the manner stated. Common iron wire may be made to give a light greater than platinum not heated. The iron becomes as hard as steel and just as elastic. Nickel is far more refractory than iron. Steel wire used in pianos becomes decarbonized, but remains hard and assumes the color of silver. Aluminum melts only at a white heat."—*Scientific American.*

---

### ARTICLE VII.

### *Pittsburgh Dental Association.*

